# Capturing the diversity of the human gut microbiota through culture-enriched molecular profiling

**DOI:** 10.1186/s13073-016-0327-7

**Published:** 2016-07-01

**Authors:** Jennifer T. Lau, Fiona J. Whelan, Isiri Herath, Christine H. Lee, Stephen M. Collins, Premysl Bercik, Michael G. Surette

**Affiliations:** Department of Biochemistry and Biomedical Sciences, McMaster University, Hamilton, ON L8S 4K1 Canada; Department of Medicine, Division of Infectious Diseases, McMaster University, Hamilton, ON L8S 4K1 Canada; Hamilton Regional Laboratory Medicine Program, Hamilton, ON L8N 4A6 Canada; Department of Medicine, Farncombe Family Digestive Health Research Institute, McMaster University, 1280 Main St W, HSC 3N-9, Hamilton, ON L8S 4K1 Canada

## Abstract

**Background:**

The human gut microbiota has been implicated in most aspects of health and disease; however, most of the bacteria in this community are considered unculturable, so studies have relied on molecular-based methods. These methods generally do not permit the isolation of organisms, which is required to fully explore the functional roles of bacteria for definitive association with host phenotypes. Using a combination of culture and 16S rRNA gene sequencing, referred to as culture-enriched molecular profiling, we show that the majority of the bacteria identified by 16S sequencing of the human gut microbiota can be cultured.

**Methods:**

Five fresh, anaerobic fecal samples were cultured using 33 media and incubation of plates anaerobically and aerobically resulted in 66 culture conditions for culture-enriched molecular profiling. The cultivable portion of the fecal microbiota was determined by comparing the operational taxonomic units (OTUs) recovered by 16S sequencing of the culture plates to OTUs from culture-independent sequencing of the fecal sample. Targeted isolation of *Lachnospiraceae* strains using conditions defined by culture-enriched molecular profiling was carried out on two fresh stool samples.

**Results:**

We show that culture-enriched molecular profiling, utilizing 66 culture conditions combined with 16S rRNA gene sequencing, allowed for the culturing of an average of 95 % of the OTUs present at greater than 0.1 % abundance in fecal samples. Uncultured OTUs were low abundance in stool. Importantly, comparing culture-enrichment to culture-independent sequencing revealed that the majority of OTUs were detected only by culture, highlighting the advantage of culture for studying the diversity of the gut microbiota. Applying culture-enriched molecular profiling to target *Lachnospiraceae* strains resulted in the recovery of 79 isolates, 12 of which are on the Human Microbiome Project’s “Most Wanted” list.

**Conclusions:**

We show that, through culture-enriched molecular profiling, the majority of the bacteria in the human gut microbiota can be cultured and this method revealed greater bacterial diversity compared to culture-independent sequencing. Additionally, this method could be applied for the targeted recovery of a specific bacterial group. This approach allows for the isolation of bacteria of interest from the gut microbiota, providing new opportunities to explore mechanisms of microbiota–host interactions and the diversity of the human microbiota.

**Electronic supplementary material:**

The online version of this article (doi:10.1186/s13073-016-0327-7) contains supplementary material, which is available to authorized users.

## Background

The gastrointestinal microbiota is a highly diverse community but the majority of the bacteria are considered unculturable since more recent sequencing-based studies have revealed greater diversity than previously detected by culture [1, 2]. Consequently, most studies characterizing the human gut microbiome have relied on culture-independent sequencing methods. These studies have provided insights into the community composition of the gut microbiota in healthy individuals [3], how it changes with environmental perturbation [4, 5], and its potential role in a variety of diseases [6, 7]. However, there are limitations to the information that can be obtained from molecular approaches alone and the isolation of organisms is required to define the roles of specific bacteria in causing or maintaining healthy and disease states. Culture also determines the viable population in a community, while most molecular methods do not distinguish between DNA obtained from live or dead cells. Furthermore, culture using selective media allows for the growth and detection of less abundant bacteria that may be missed by insufficient sequencing depth in culture-independent studies [8].

A few recent studies have attempted to characterize the culturable human gut microbiota by combining culture with next-generation sequencing [9] but only a maximum of 50 % of the operational taxonomic units (OTUs) detected by 16S rRNA gene sequencing of fecal samples were cultivable [10, 11]. Lagier et al. [8] developed a culturomics method, which used 212 conditions for the cultivation of the fecal microbiota. However, bacterial colonies were identified with MALDI-TOF mass spectrometry, making it difficult to directly compare to OTUs obtained from pyrosequencing in order to determine the culturable proportion of the microbiota [8]. This study identified 174 novel bacterial species, demonstrating that culture is still a valuable method for exploring the gut microbiome. Another study, by Rettedal et al. [11] tested several culture conditions to capture a representative proportion and maintain the overall community structure of the gut microbiota. From ten media, 88 % of family level taxonomic groups were recovered and, interestingly, 40 % more OTUs were found by culture than by culture-independent methods, demonstrating the potential advantages of culture for capturing microbial diversity in the gut. Another study has shown that personalized culture conditions, although only representing 50 % of the phylotypes observed from culture-independent sequencing, could colonize germ-free mice in a similar manner to the complete fecal samples [10].

**Fig. 1:**
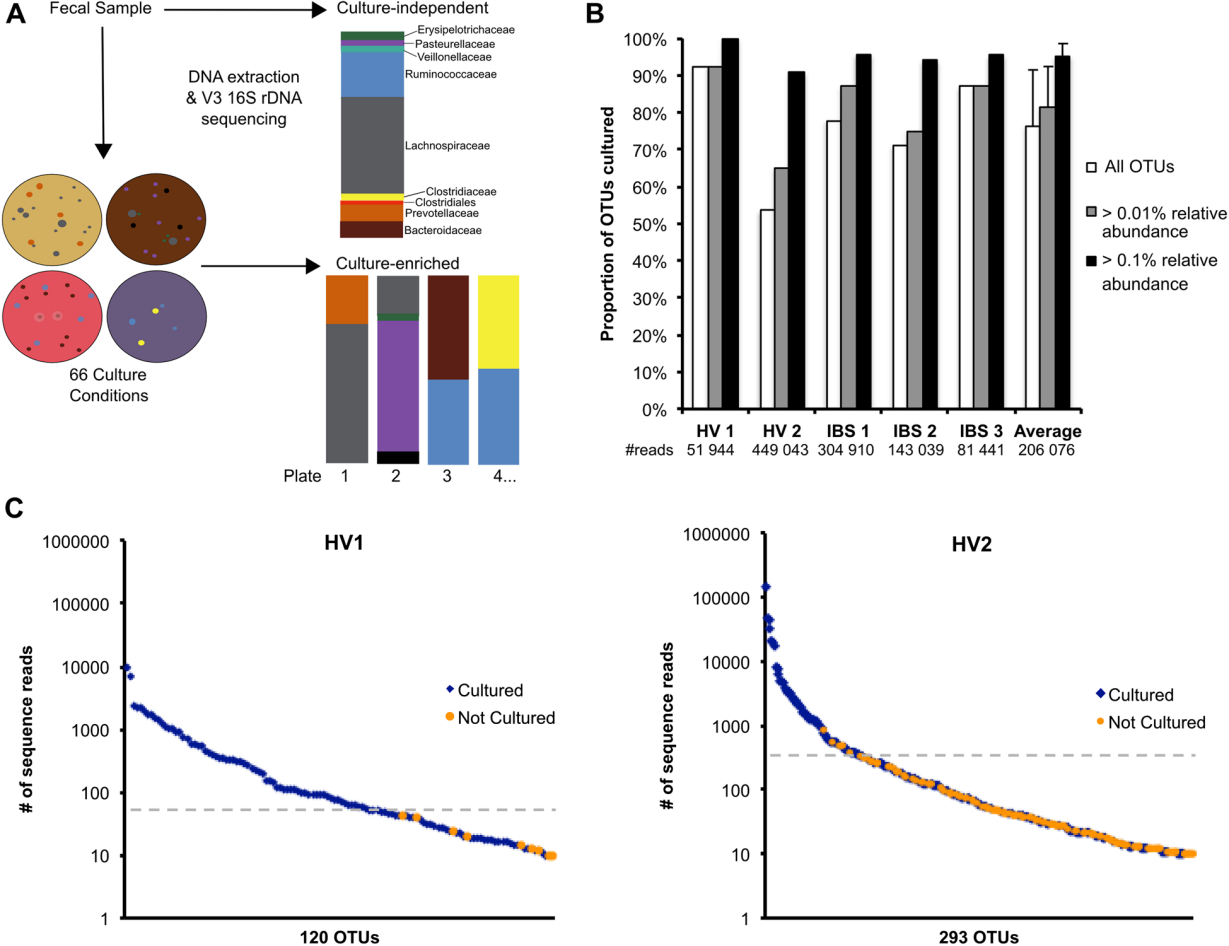
Culture-enriched molecular profiling captures the majority of OTUs. **a** The culture-enriched molecular profiling protocol. Fecal samples were cultured using 66 conditions and colonies were harvested from each plate. 16S rRNA gene sequencing of DNA extracted from colonies was compared with sequencing of the fecal sample. **b** OTUs from 16S rRNA gene sequencing of culture-enriched fecal samples were compared with those from culture-independent sequencing. The number of reads in the culture-independent sequencing of each sample is below the *x-axis*. The average proportion of OTUs cultured from each sample is shown; *error bars* represent standard deviation. **c** All OTUs from culture-independent sequencing of HV1 and HV2 samples were ranked by abundance and compared with OTUs detected by culture-enriched sequencing. Each *point* represents one OTU and the *dashed line* indicates 0.1 % relative abundance in the culture-independent sequencing. *HV* healthy volunteer, *IBS* irritable bowel syndrome

In this study, a method of culture-enriched molecular profiling [12] which combines extensive bacterial culture with 16S rRNA gene sequencing (Fig. 1a) was used to investigate the proportion of the microbiota that was readily cultured from the gastrointestinal tract. Using 66 culture conditions and applying the same 16S rRNA gene sequencing method to both the cultured community and the fecal samples, we demonstrate that the majority of OTUs could be detected through culture-enriched molecular profiling and culture detected greater diversity than culture-independent methods. Culture-enriched molecular profiling was further applied to the targeted culturing of *Lachnospiraceae* isolates.

## Methods

### Sample collection

This study was approved by the Hamilton Integrated Research Ethics Board and donors provided consent prior to participation. Healthy volunteers (HV1–7) had no gastrointestinal (GI) symptoms and did not use antibiotics within 6 months of the study. Irritable bowel syndrome (IBS) patients (IBS1–4) were diagnosed as diarrhea-predominant or mixed subtype based on Rome III criteria [13] and recruited from the GI Clinical Investigation Unit at McMaster University.

Fresh fecal samples were transferred to sterile specimen containers immediately following defecation and stored in airtight bags containing an anaerobic pouch (Oxoid, UK) and ice-pack until transfer to an anaerobic chamber (5 % CO_2,_ 5 % H_2_, 90 % N_2_; Shel Labs, Cornelius, OR, USA), which occurred within 1–5 h of collection. Afterwards, all work was completed inside an anaerobic chamber. Samples were mechanically mixed with a sterile spatula and cultured as described below.

### Culturing of fecal samples

Inside an anaerobic chamber, 0.1 g of fecal sample was diluted in 900 μl of pre-reduced brain heart infusion (BHI) broth (BD, Sparks, MD, USA) with 0.05 % L-cysteine hydrochloride hydrate (10^0^ dilution). We plated 100 μl of 10^−3^ and 10^−5^ dilutions on pre-reduced 100-mm agar plates (media types listed below). One set of media was incubated at 37 °C for 5 days in an anaerobic chamber and another set was incubated at 37 °C in 5 % CO_2_ for 3 days. After incubation, colonies were collected from each plate by adding 1 ml BHI broth and scraping the surface of plates with a cell scraper. Both dilutions (10^−3^ and 10^−5^) of each media were combined and 500 μl of the harvested colonies was frozen in 10 % skim milk at −80 °C as stocks and 500 μl was used for DNA extraction as described below.

Media used in this study included (Additional file 1: Table S1): brain heart infusion (BHI) agar (BD), BHI + 0.5 g/L L-cysteine hydrochloride hydrate, 10 mg/L hemin, and 1 mg/L vitamin K (supplement set A), BHI + 10 mg/L colistin sulphate and 5 mg/L naladixic acid (supplement set B), BHI + supplement set A and B, BHI + supplement set A and 20 mg/L gentamycin, BHI + supplement set A and 1 % propionic acid, 0.2× BHI or M9 minimal media (BD) + one of 1 g/L inulin, 0.5 g/L pectin, 0.5 g/L cellulose, 0.5 g/L mucin, or 0.5 g/L starch, Bifidobacterium Selective Media (Fluka, St. Louis, MO, USA), Gut Microbiota Medium [10], phenylethyl alcohol agar (BD) + 5 % sheep’s blood (Cedarlane, Canada), Bacteroides Bile Esculin agar [14], Actinomyces Isolation agar (BD), Columbia blood agar (BD) + 5 % sheep’s blood, colistin naladixic agar (BD) + 5 % sheep’s blood, McKay agar [15], chocolate agar (BD), tryptic soy agar (BD) + 5 g/L yeast extract (BD) + supplement set A, fastidious anaerobe agar (Neogen, Lansing, MI, USA), deoxycholate agar [14], MacConkey agar (BD), kanamycin vancomycin laked blood agar [14], mannitol salt agar (BD), de Man Rogosa Sharpe agar (BD), and cooked meat broth (Fluka) + 1.2 % agar. All media were prepared following the manufacturer’s instructions or as previously described unless otherwise mentioned.

For experiments showing that cultured communities were represented by viable organisms (Additional file 2: Figure S4), 100 mg of frozen IBS1 sample was diluted 1:10 with 70 % isopropanol and heated to 70 °C for 1 min to sterilize (“no-growth” samples) or DNA extracted from IBS1 sample was used (DNA samples). The samples were diluted to 10^−5^ with BHI with 0.05 % L-cysteine as described above and cultured anaerobically on phenylethyl alcohol agar (PEA) and M9 + cellulose plates for 1 h at 37 °C before plates were scraped and DNA was extracted as described below.

For the comparison of fresh and frozen samples, one aliquot was immediately cultured and another was stored at −80 °C for up to one year (IBS1 and HV2 for 4 months, IBS3 for 1 year). To compare anaerobic and aerobic samples, fecal samples were divided during collection; one portion was stored with an anaerobic pouch at 4 °C until culturing and the other was stored at 4 °C without an anaerobic pouch (aerobic sample).

### DNA extraction and 16S rRNA gene sequencing

DNA extraction and purification were performed as previously described [16]. Briefly, 500 μl of harvested colonies or 0.1 g of fecal sample was mechanically homogenized with 0.2 g of 0.1 mm glass beads (MoBio, Carlsbad, CA, USA; an additional 0.2 g of 2.8 mm glass beads was added to fecal samples) in 800 μl of 200 mM NaPO_4_, pH 8 and 100 μl guanidine thiocyanate-EDTA-*N*-lauroyl sarcosine. Enzymatic lysis with 50 μl lysozyme (100 mg/ml), 50 μl mutanolysin (10 U/μl), and 10 μl RNase A (10 mg/ml) for 1 h at 37 °C was followed by the addition of 25 μl 25 % sodium dodecyl sulfate (SDS), 25 μl Proteinase K (20 mg/ml,) and 75 μl 5 M NaCl and further incubated for 1 h at 65 °C. Supernatants were collected and DNA extracted with phenol-chloroform-isoamyl alcohol (25:24:1; Sigma, St. Louis, MO, USA) and further purified using DNA Clean and Concentrator-25 columns (Zymo, Irvine, CA, USA) as per the manufacturer’s instructions. Isolated DNA was stored at −20 °C.

PCR amplification of the V3 region of the 16S rRNA gene was performed as previously described [17] with the following modifications: a 60 μl reaction containing 1.25 mM MgCl_2_, 2.5 mM of each dNTP, 100 nM of each barcoded primer, and 1.25 U Taq was divided into 3 × 20 μl reactions for amplification. PCR conditions consisted of an initial denaturation at 94 °C for 2 min, 30 cycles of 94 °C for 30 s, 50 °C for 30 s, 72 °C for 30 s, followed by a final elongation at 72 °C for 10 min. Purified PCR products were sequenced using the Illumina MiSeq platform by the McMaster Genome Facility (Hamilton, ON, Canada).

### Sequence processing and analysis

16S rRNA gene sequence processing was completed as previously described [16]. In brief, Illumina sequence reads were trimmed to the forward and reverse primers of the V3 region with Cutadapt [18] and paired-end sequences were aligned with PANDAseq [19]. OTUs were binned at 97 % similarity using AbundantOTU [20] and taxonomy was assigned using the Ribosomal Database Project (RDP) classifier [21] against the Greengenes reference database (4 February 2011 release) [22] using Quantitative Insights Into Microbial Ecology (QIIME) [23]. Unassigned OTUs and singletons were not included. The total number of reads for the culture-enrichment experiment was 30,581,472. For analysis of the culture-enriched experiments, only OTUs with at least ten sequence reads were included and considered cultured since the number of reads between replicates was less reproducible below this depth (data not shown).

**Fig. 2:**
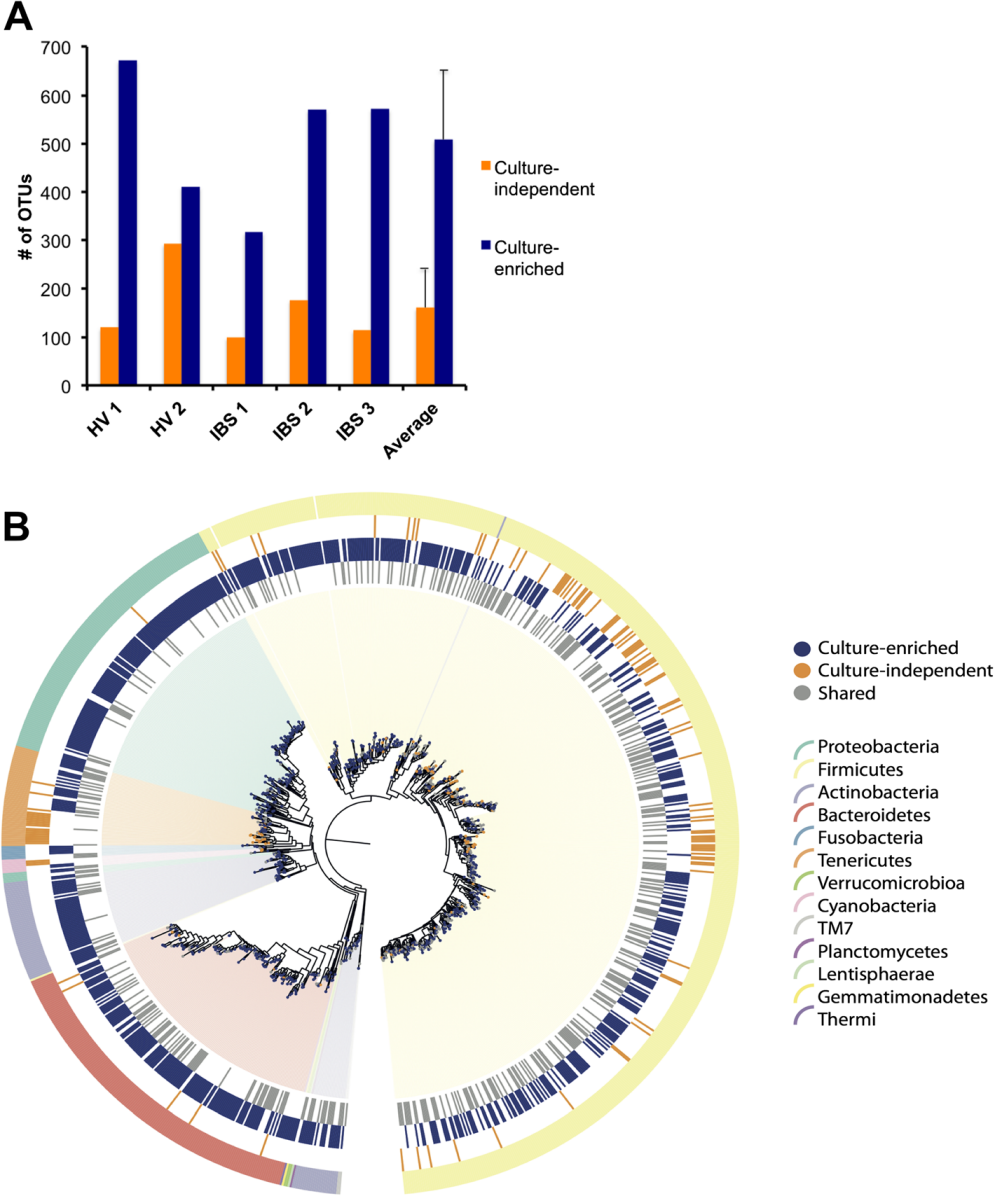
Culture-enriched molecular profiling detects more OTUs than culture-independent sequencing. **a** A comparison of the number of OTUs detected by culture-independent and culture-enriched sequencing for each fecal sample. The average number of OTUs detected by each method is shown; *error bars* represent standard deviation. **b** Phylogeny of Greengenes 16S rRNA gene sequences to which OTUs were assigned (clustered to 97 % sequence similarity). The *branch nodes* and three *innermost rings* are labeled based on detection by culture-independent, culture-enriched, or both methods. The *outer ring* and *background* are colored by phyla, as indicated in the *legend*

Relative abundance taxonomic summaries, beta diversity, and rarefactions were completed with QIIME. The 16S rRNA gene phylogeny depicting OTUs from culture-independent, culture-enriched, or both methods (Fig. 2b) was created by pruning the 4 February 2011 release of the Greengenes phylogeny clustered to 97 % similarity. This phylogeny was pruned using QIIME and visualized using GraPhlAn v0.9.7 [24].

### *Lachnospiraceae* isolation and identification

Freshly collected HV7 and IBS4 fecal samples were cultured anaerobically on BHI + 1 g/L inulin (BHI + inu) and cooked meat agar (BEEF). We streaked 146 colonies for purity and single colonies were boiled in 5 % Chelex for 15 min to lyse cells. Colony PCR to amplify the 16S rRNA gene was performed using 8f (5′-AGAGTTTGATCCTGGCTCAG-3′) and 927r (5′-CCGTCAATTCCTTTRAGTTT-3′) primers. PCR conditions consisted of an initial denaturation at 94 °C for 2 min, 32 cycles of 94 °C for 1 min, 56 °C for 1 min, 72 °C for 2 min, followed by a final elongation at 72 °C for 10 min. PCR products were Sanger sequenced by Beckman Coulter Genomics (Danvers, MA) using the 8f primer. Taxonomic assignment of isolates was made with the online RDP Classifier [21] using 16S rRNA training set 10 with a confidence threshold of 80 %. 16S rRNA gene sequences of the isolates were compared against the *Lachnospiraceae* consensus sequences from the Human Microbiome Project’s (HMP) “Most Wanted” list (obtained on 18 November 2014) using Megablast in Geneious v5.6.4 [25].

### *Lachnospiraceae* phylogeny

The 16S rRNA sequence of 107 *Lachnospiraceae* type isolates obtained from RDP [21] (on 12 November 2014) were aligned using MUSCLE v3.8.31 [26] (Edgar, 2004). jModelTest v2.1.1 [27] was used to determine that the data most appropriately fit the generalized time-reversible (GTR) model with invariable sites and a discrete gamma distribution. A maximum likelihood molecular phylogeny of these isolates was obtained using raxmlGUI v1.3.1 [28] implementing RAxML v8.0.26 [29] with 100 bootstrap replicates. pplacer v1.1 [30] was used to add the 16S rRNA gene sequences of 79 cultured *Lachnospiraceae* isolates, obtained as described above. The resulting phylogeny was visualized and midpoint rooted using FigTree v1.4.0 [31].

## Results

In this study, the cultivable proportion of the human gut microbiota was determined from the fecal samples of two healthy donors and three patients with irritable bowel syndrome (IBS). IBS samples were included since the culturable bacterial community may be affected by health status and IBS is a common intestinal disorder in which the microbial composition of the gut is altered and implicated in the pathogenesis of the disorder [32]. Using a method of culture-enriched molecular profiling [12], anaerobic fecal samples were cultured using 33 media and incubation of plates anaerobically and aerobically resulted in 66 culture conditions (Fig. 1a). The media used were comprised of commercially available components, without the requirement of complex additions like rumen fluid or fecal extracts used in previous studies [8, 33], and were chosen based on previous culture-enriched molecular profiling studies, with additional media added for specific cultivation of the gut microbiota, including Bacteroides Bile Esculin, Bifidobacterium Selective media, Gut Microbiota medium [10], and the addition of prebiotics and resistant carbohydrates [33] (Additional file 1: Table S1). The cultivable portion of the fecal microbiota was determined by pooling all colonies from each type of media (per fecal sample) and performing 16S rRNA gene sequencing on each plate pool individually, referred to as culture-enriched molecular profiling. A portion of the pooled colonies was reserved for future bacterial isolation. Since the same DNA extraction and sequencing methods were used for both the cultured plate pools and the fecal sample, the OTUs from the culture could be directly compared with the OTUs from culture-independent sequencing of the original fecal sample to determine which OTUs were cultured on each type of medium.

### Culture-enriched molecular profiling detects the majority of OTUs in fecal samples

An average of 76 % of all OTUs observed in culture-independent sequencing of the fecal samples was also detected by culture-enrichment (Fig. 1b). The largest proportion of OTUs cultured was 93 % from the healthy volunteer 1 (HV1) sample. No obvious differences were observed in the proportions of OTUs cultured between IBS and healthy samples (an average of 79 and 73 % of all OTUs were cultured, respectively). Using thresholds of 0.01 and 0.1 % (in the culture-independent sequencing), an average of 81 and 95 % of OTUs were cultured, respectively (Fig. 1b), indicating that culture-enriched molecular profiling could detect the majority of the abundant members of the fecal community. A comparison of several OTU picking methods, including AbundantOTU [20] and UCLUST [34] against different reference databases, resulted in similar proportions cultured for each sample, indicating that these results were robust to the method of OTU clustering (Additional file 2: Figure S1). However, the proportion of OTUs cultured was affected by culture-independent sequence depth; as the number of culture-independent sequence reads increased, the percent of OTUs cultured decreased (Additional file 2: Figure S2). Shallow sequencing depth (< 5000 reads) in culture-independent sequencing, as used in previous studies [10], would artificially inflate the proportion of the cultured microbiota due to incomplete culture-independent sampling of fecal samples. For each sample, ranking the OTUs from culture-independent sequencing by abundance reveals a few highly abundant OTUs and a long tail distribution of lesser abundant OTUs, as expected in human gut microbiota communities [35] (Fig. 1c; Additional file 2: Figure S3). Above 0.1 % relative abundance, all OTUs from HV1 were recovered by culture and only five OTUs from HV2 were not cultured (Fig. 1c). Uncultured OTUs were of low abundance (< 0.8 % relative abundance) in the culture-independent sequencing. Twelve OTUs with relative abundances greater that 0.1 % were not cultured from the donor samples and included *Cyanobacteria*, *Clostridia*, *Mollicutes*, and *Bacteroidetes*. However, 9 of these 12 OTUs were detected by culture in the other fecal samples.

Several bacterial genera were present at less than 1 % abundance in culture-independent sequencing but recovered by culture at higher abundances on plates (Additional file 2: Figures S4e and S5), thus demonstrating the advantages of culture for recovering low abundance bacteria from the GI microbiota. Comparison of each plate community to the culture-independent fecal samples showed that no single medium accurately represented the composition of the fecal sample, indicating that OTUs detected by culture represent viable organisms and not DNA deposited on plates (Additional file 2: Figure S4a–d). To further demonstrate this, a sterilized fecal sample from the IBS1 donor and DNA extracted from the same sample were plated on two media, representing cultured communities most similar and most different to the composition of the fecal sample (Additional file 2: Figure S4b–d). The composition of the no-growth and DNA controls were very different from either the fecal sample or the pooled plate. Indeed, the negative growth controls from both types of media were most similar to each other, providing evidence that 16S sequencing of plates represent growth of viable cells and not DNA or dead cells deposited from the fecal sample. Goodman et al. [10] have previously shown that sequencing of pooled colonies cultured from a fecal sample diluted 10,000-fold resulted in less than 2 % of the sequences being derived from non-viable cells.

### The minimal conditions for culture-enrichment reflects the inter-individual heterogeneity of the human gut microbiota

The cultured communities on each media differed between samples, reflecting the inter-individual heterogeneity of the microbiota [3]; thus, a minimal set of media that could capture the majority of OTUs in all samples would be difficult to predict, similar to results previously reported for the airway microbiome [12]. By determining the minimum culture conditions needed to recover all culturable OTUs present at greater than 0.1 % in the fecal samples, we observed that the set of culture conditions required was different for all five fecal samples (Additional file 2: Figure S6). Interestingly, 23 of the 33 anaerobic culture conditions were required across the five samples, indicating that most of the media used were non-redundant, and additional fecal samples may necessitate the use of the other media. There was a distinct difference in the bacterial communities recovered from anaerobic incubation compared with aerobic growth (Additional file 2: Figure S5); *Escherichia* was the most abundant OTU on the majority of aerobic media used so future studies could reduce the number of aerobic conditions.

### The cultured community is affected by storage conditions of the fecal sample

Since immediate culturing of anaerobic fecal samples may not always be feasible, we determined the affect of freezing at −80 °C or exposure to ambient oxygen on the culturable fecal community. Fresh, anaerobic fecal samples were cultured as done previously and the culture-enriched profiles were compared with those from fecal samples that were frozen or exposed to oxygen (Additional file 2: Figure S7a–e). All conditions tested showed that the cultured microbiome profile was different after freezing, with Bray–Curtis dissimilarity distances greater than 0.3 (Additional file 2: Figure S7a, d), while fecal samples were differentially affected by aerobic exposure, with Bray–Curtis dissimilarity distance ranging from 0.15 to 0.65 (Additional file 2: Figure S7b, c, e). Since the compositions of the fecal samples were different from each other and each medium used selected for different bacteria, no abundant bacterial groups were identified as consistently altered after storage. Optimal recovery of bacteria required stool samples to be kept anaerobic immediately after collection and plated without freezing, as exposure to oxygen and storage at −80 °C altered the culturable community.

### Culture-enriched molecular profiling captures greater bacterial diversity than culture-independent sequencing

Importantly, we observed that culture-enrichment recovered more OTUs than culture-independent sequencing. Taking into account all OTUs detected in the five samples by both methods, 1051 OTUs (67 % of the total) were detected only by culture-enrichment, 390 OTUs (25 %) were found by both methods, and 118 OTUs (8 %) were detected only by culture-independent sequencing. For each sample, more OTUs were observed by culture-enriched sequencing than culture-independent sequencing (Fig. 2a). This suggests that even with deep sequencing of a fecal sample (average depth = 206 076 reads), culture detects greater bacterial diversity compared with culture-independent methods. The OTUs that were detected only by culture were mainly distributed across six phyla: *Actinobacteria*, *Bacteroidetes*, *Firmicutes*, *Fusobacteria*, *Proteobacteria*, and *Tenericutes*. These low abundance organisms may include bacteria from the colonic mucosa and other sites in the GI tract, where they may be present at higher local abundance but at too low abundance in stool to be detected by the culture-independent sequencing depths used in this study. OTUs that were culture-only or detected by both methods were evenly distributed across the bacterial taxonomy (Fig. 2b), indicating that cultured OTUs represent members of the fecal microbiota and not plate contamination. Uncultured OTUs included a *Tenericutes* branch and a few *Firmicutes* clades.

### Targeted culturing of *Lachnospiraceae* isolates

**Fig. 3:**
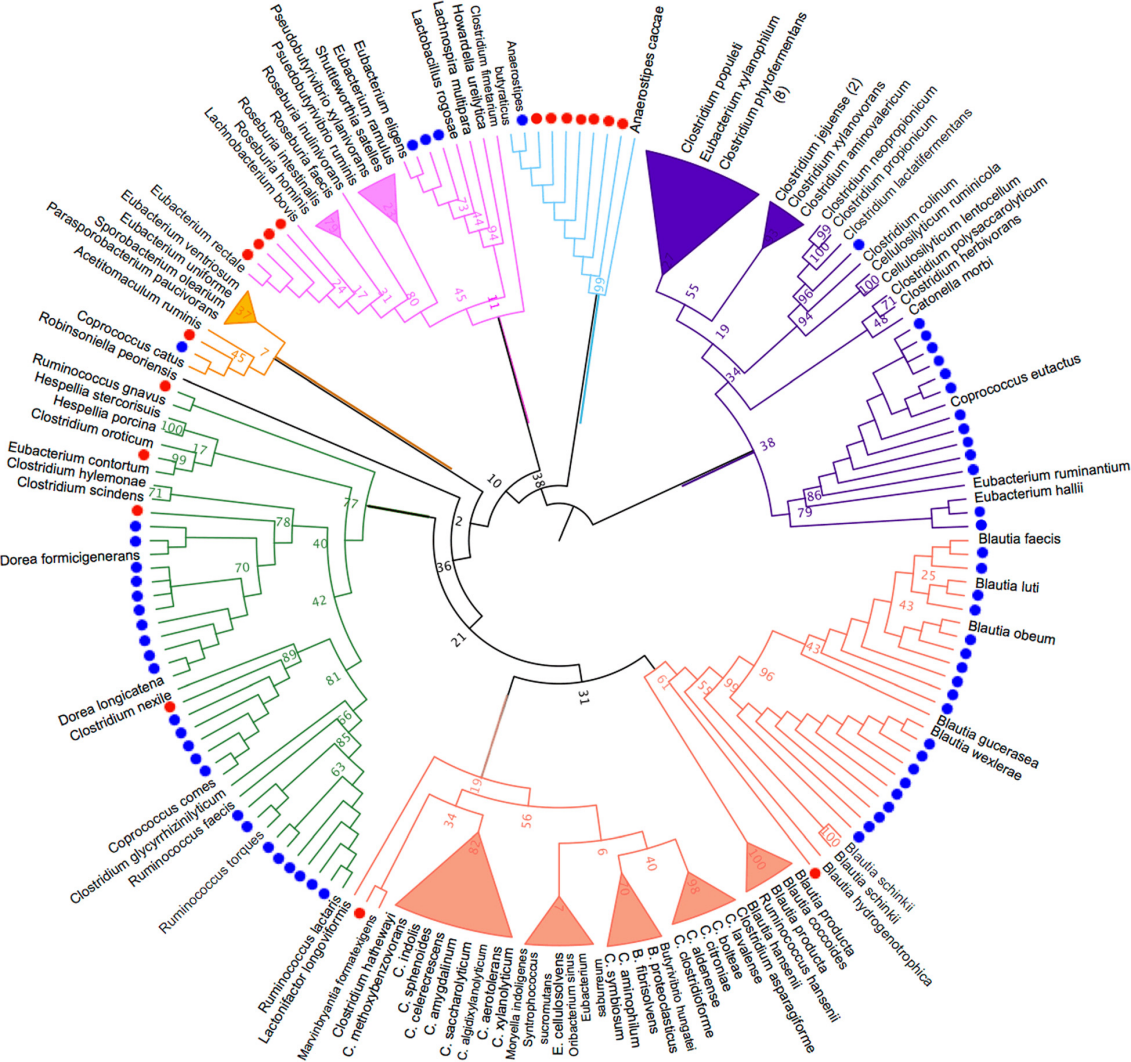
16S rRNA maximum-likelihood tree of 79 *Lachnospiraceae* isolates in relation to 107 RDP *Lachnospiraceae* type isolates. V1–V4 16S rRNA gene sequences of cultured *Lachnospiraceae* isolates were placed into a phylogeny created with RDP *Lachnospiraceae* type isolates. *Blue* represents cultured isolates; *red* represents cultured isolates with < 97 % similarity to RDP reference isolates. *Branch labels* show bootstrap values. *Branch colors* highlight clades for visual aid. *C Clostridium*, *B Butyrivibrio*, *E Eubacterium*

Culture-enriched molecular profiling was applied to additional healthy (HV3) and IBS (IBS4) fecal samples for the targeted culturing of *Lachnospiraceae* isolates to demonstrate that a bacterial group of interest could be isolated from fecal samples, when the bacterial composition was not known, by using results of culture-enriched profiling of previous samples (Additional file 2: Figure S5). There is extensive *Lachnospiraceae* diversity in the human gut microbiota but this prevalent and abundant family is poorly represented by reference genomes [36]. From analysis of the culture-enriched profiling of previous fecal samples, it was determined that communities grown on anaerobic BHI + inu and BEEF agar were dominated by *Lachnospiraceae* (Additional file 2: Figure S5). Consistent with our culture-enrichment results, inulin has been shown to increase growth of butyrate-producing bacteria, including *Lachnospiraceae*, both in vivo [37] and in vitro [38]. HV3 and IBS4 fecal samples were cultured anaerobically on BHI + inu and BEEF and colonies were isolated (as opposed to plate pooling). Sanger sequencing of the 16S rRNA gene of 146 isolated colonies resulted in the identification of 79 *Lachnospiraceae* isolates, including representatives of the genera *Blautia*, *Marvinbryantia*, *Ruminococcus*, *Dorea*, *Eubacterium*, *Anaerostipes*, *Clostridium*, and *Coprococcus* (Additional file 3: Table S2). Additionally, 18 isolates had less than 97 % similarity to Ribosomal Database Project (RDP) reference isolates at the genus level [21], as supported by the phylogenetic distance of these isolates to the 107 RDP *Lachnospiraceae* reference sequences (Fig. 3). These 18 isolates may be novel species not currently represented by the RDP type isolates. Comparison of the 16S rRNA gene sequences of the *Lachnospiraceae* isolates to the Human Microbiome Project’s (HMP) Most Wanted taxa resulted in a match to 12 sequences with 100 % identity, including four organisms of medium priority (Additional file 4: Table S3). The “Most Wanted” taxa represent uncultured but abundant and prevalent organisms from the HMP 16S rRNA gene sequencing data set [39]. Isolation of novel representatives of the gut microbiome and whole genome sequencing of these organisms will add critical information to reference genome collections and lead to more accurate assignments for metagenomics, transcriptomics, and proteomic studies.

## Discussion

Although other studies have attempted to culture the gut microbiota using complicated methods such as chemostats [40] and microfluidic devices [41], we show that the use of extensive culture conditions and simple agar plates results in the comprehensive cultivation of bacteria from the gut microbiota. Using significantly fewer and less complex culture conditions than Lagier et al. [8], we were still able to capture the majority of OTUs seen by culture-independent sequencing. Indeed, we believe that this is the first study showing that > 50 % of the OTUs from a fecal sample can be recovered by culture. We also show that culture revealed greater bacterial diversity than detected by culture-independent sequencing. These organisms, absent or underrepresented in culture-independent profiling, may contribute to host phenotypes so methods to detect and isolate this population are critical for future studies. We did not observe a difference in the proportion of OTUs that could be cultured between IBS and healthy fecal samples; however, our sample size was small and further studies should be carried out to confirm this. By applying the data generated from culture-enriched molecular profiling in the first part of this study, we were able to carry out the targeted isolation of *Lachnospiraceae*. This resulted in the recovery of up to 18 species not represented by a reference database from culturing only two fecal samples on two media conditions, demonstrating that culture is valuable for accessing novel members of the gut microbiota.

Although most fecal OTUs were cultured in this study, some OTUs likely contained more than one strain and some strains may have been missed by culture; metagenomics may sort this out. Some OTUs were detected only by culture-enrichment or culture-independent sequencing, suggesting that both methods are complementary for studying the gut microbiota since the combination is more sensitive than either method alone. The uncultured OTUs may have been missed due to the following reasons: stochastic sampling if the bacteria were rare in the sample, DNA could have originated from dead cells, slower growing organisms may have been out-competed by less fastidious members of the community, or the media used in this study may have been missing a nutrient required for growth or prevented growth of a bacterial partner critical for survival. This may also explain why some OTUs were not detected by culturing in one fecal sample but recovered from another donor.

Consistent with previous studies [8, 11], we observed many OTUs that were only detected by culture. Certain bacterial groups may be under-represented by primers used in 16S sequencing of complex communities, such as stool, since poor primer alignment would result in these sequences being outcompeted during PCR of mixed community DNA [36]. However, these bacteria can be grown using selective media and subsequently detected by culture-enriched molecular profiling since there may be less competing DNA in 16S rDNA amplification of the plate pools. As previously reported, bacteria present at < 10^6^ cells per gram of feces is likely to be missed by sequencing depths used in most microbiome studies [8], but the ability to grow less abundant bacteria on media would increase recovery of DNA for sequencing these rarer members of the population. Additionally, DNA from bacterial spores in stool may be underrepresented by 16S sequencing, depending on the method of DNA extraction [42], but germination of spores during culture would allow for increased detection of spore-forming populations.

Culture-enriched molecular profiling for the targeted recovery of specific bacterial groups will be a powerful tool for the study of the gut microbiota since bacteria of interest could be recovered after sequencing has revealed which media support its growth, as represented in Additional file 2: Figure S5. Furthermore, since this method allows for the development of a bacterial community on a plate, it does not exclude syntrophic interactions that may be required for growth of some bacteria [43]. In addition, subsequent culturing of plate pools using combinations of antibiotics and other selective media components can be used to develop more refined culture conditions for specific bacteria [11]. The application of this method for the recovery of novel representatives of the gut microbiota will add to reference databases and lead to more accurate assignment for future “omic” studies. To advance the microbiome field beyond correlative investigations and to test hypotheses generated from culture-independent studies, it is critical to isolate bacteria of interest from the gut microbiota and culture-enriched molecular profiling allows for such isolation.

## Conclusions

We demonstrate that the majority of the human gut microbiota can be captured by culture-enriched profiling and this method can be applied to the recovery of specific bacterial groups. This method also highlights the ability of culture for recovering low abundance bacteria and for revealing diversity that may be underrepresented by other methods. Isolation of bacteria will allow us to explore the therapeutic potential of bacterial products which may directly affect the host or microbial community. Access to the cultured human microbiota will offer detailed functional characterization of bacteria and will facilitate the discovery of their biological activities during host–bacteria and inter-bacterial interactions in health and disease.

## Abbreviations

BEEF, cooked meat agar; BHI, brain heart infusion agar; GI, gastrointestinal; HMP, Human Microbiome Project; HV, healthy volunteer; IBS, irritable bowel syndrome; OTU, operational taxonomic unit; QIIME, Quantitative Insights Into Microbial Ecology; RDP, Ribosomal Database Project.

## Additional files

Additional file 1: Table S1. List of media used for culture-enriched molecular profiling. (XLSX 30 kb) Additional file 2: Figure S1. Comparison of the effect of OTU picking method on the proportion of OTUs cultured. Figure S2 Comparison of effect of sequencing depth on the proportion of OTUs cultured. Figure S3 Cultured and uncultured OTUs ranked by abundance for IBS1–3 samples. Figure S4 Comparison of cultured communities with culture-independent sequencing of fecal samples. Figure S5 Heat map of family-level taxa abundances in culture-enriched and culture-independent sequencing. Figure S6 Culture conditions required to capture the most abundant OTUs from each fecal sample Figure S7 Culturing fresh fecal samples compared with frozen samples and anaerobic fecal samples compared with aerobic samples. (DOCX 2200 kb) Additional file 3: Table S2. Classification of *Lachnospiraceae* isolates cultured from Donor HV7 and IBS4 fecal samples. (XLSX 38 kb) Additional file 4: Table S3. HMP Most Wanted matches of *Lachnospiraceae* isolates from HV7 and IBS4 samples. (XLSX 59 kb)
