# Supplementary material for: Capturing the diversity of the human gut microbiota through culture-enriched molecular profiling
Source: Genome Med. 2016 Jul 1;8:72. doi: 10.1186/s13073-016-0327-7 (PMC4929786; doi:10.1186/s13073-016-0327-7)
Supplement: Additional file 2: Figure S1. — Comparison of the effect of OTU picking method on the proportion of OTUs cultured. Figure S2 Comparison of effect of sequencing depth on the proportion of OTUs cultured. Figure S3 Cultured and uncultured OTUs ranked by abundance for IBS1–3 samples. Figure S4 Comparison of cultured communities with culture-independent sequencing of fecal samples. Figure S5 Heat map of family-level taxa abundances in culture-enriched and culture-independent sequencing. Figure S6 Culture conditions required to capture the most abundant OTUs from each fecal sample Figure S7 Culturing fresh fecal samples compared with frozen samples and anaerobic fecal samples compared with aerobic samples. (DOCX 2200 kb) [file 13073_2016_327_MOESM2_ESM.docx]

**Supplementary Figures**

**Figure S1. Proportion of OTUs culture is not dependent on OTU picking method.** OTUs from 16S rRNA gene sequencing of culture-enriched fecal samples were compared to culture-independent sequencing. OTUs were picked using AbundantOTU, or Uclust using Greengenes 2011, Greengenes 2013 or SILVA as reference databases as seeds.

**
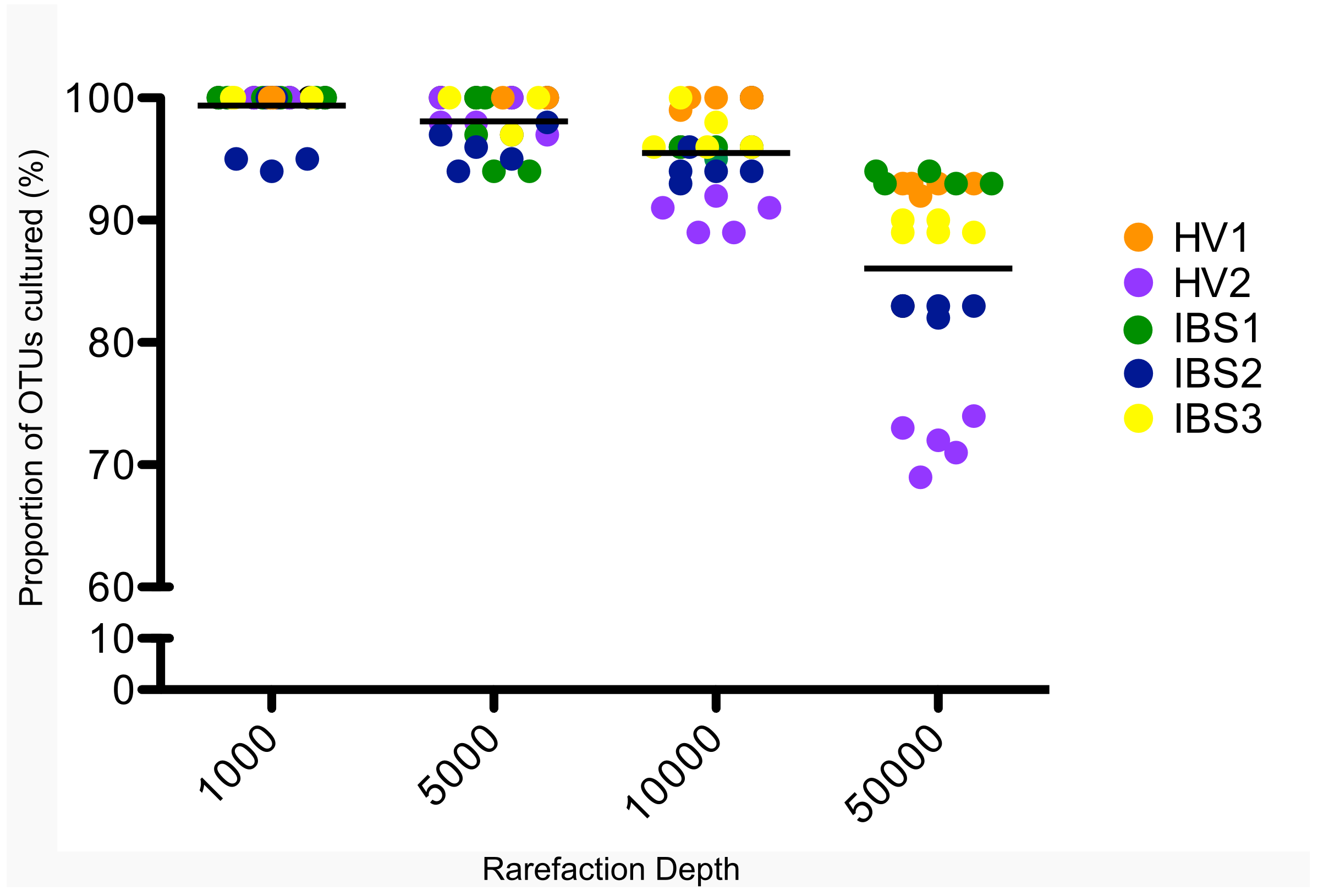
**

**Figure S2.** **Proportion of OTUs cultured is dependent on culture-independent sequencing depth.** Samples were rarified to 1 000, 5 000, 10 000 and 50 000 sequence reads to determine, *in silico,* the proportion of OTUs that would be cultured at each experimental sequencing depth. Each rarefaction was performed five times per sample, each represented as one point. Lines indicate the average proportion of OTUs cultured at each rarefaction depth.

**
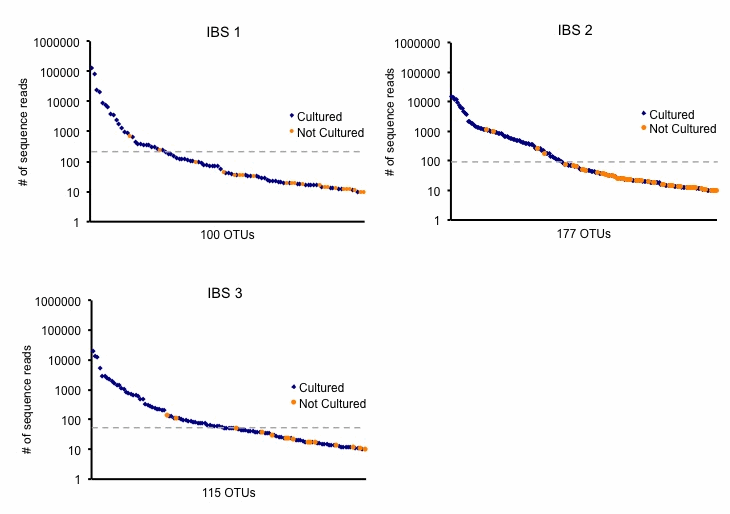
**

**Figure S3. The majority of the uncultured OTUs are low abundance in culture-independent sequencing of the fecal samples.** All OTUs from culture-independent sequencing of fecal samples from IBS 1-3 donors were ranked by abundance and compared to OTUs detected by culture-enriched sequencing to determine if each OTU was cultured or not cultured. Each point represents one OTU and the dotted line indicates a cutoff of 0.1% abundance in the culture-independent sequencing. The number of OTUs in each sample is indicated on the x-axis.

**
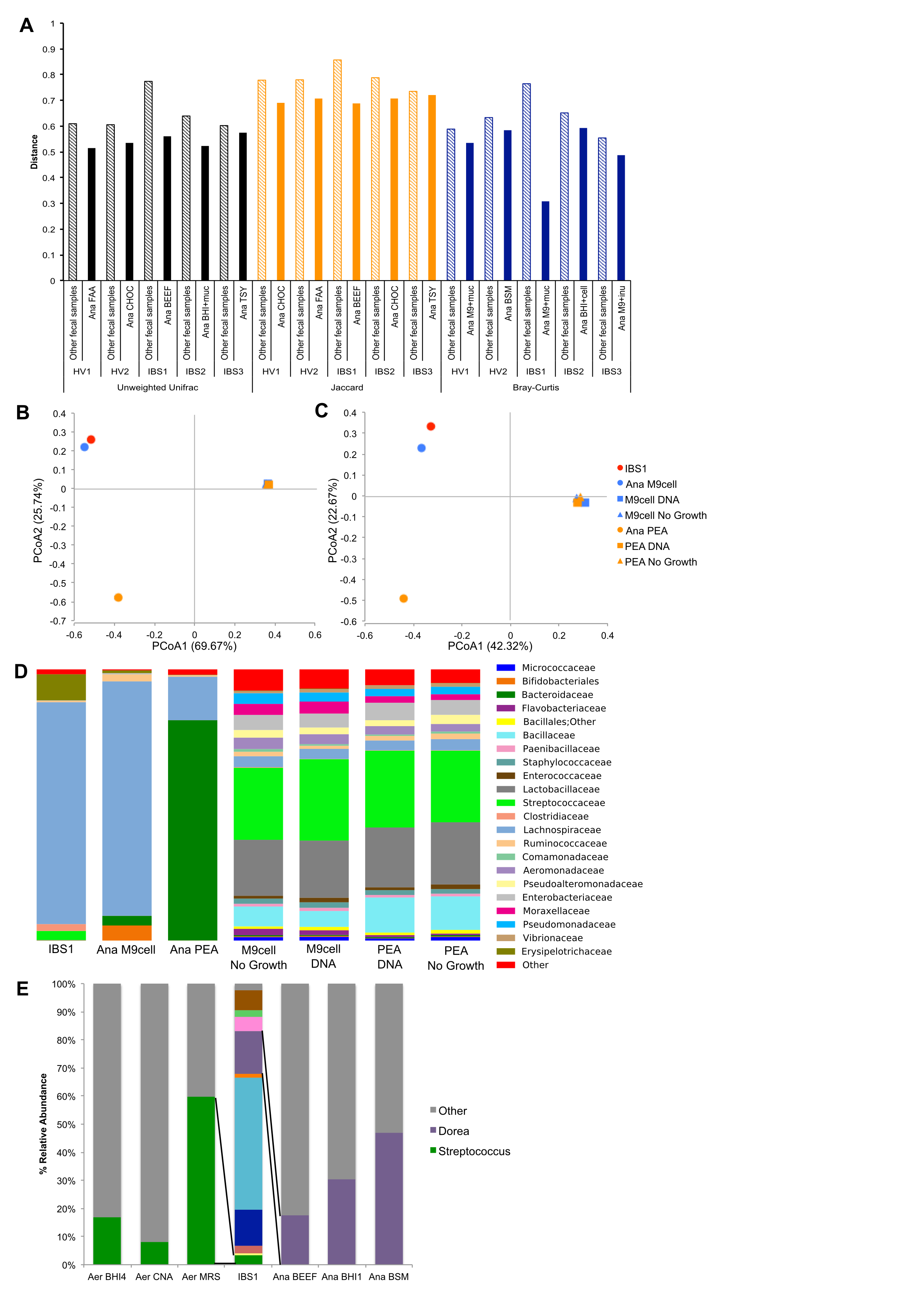
**

**Figure S4.** **Cultured communities are not similar to fecal samples.** A) Unweighted Unifrac, binary Jaccard and Bray-Curtis distances of culture-independent sequencing of 5 samples compared to their most similar cultured community (solid bars). For comparison, the distance between each fecal sample and samples from the other 4 donors is shown (hatched bars). Samples were rarified to 2900 sequences. B) Bray-Curtis PCoA, C) Unweighted Unifrac PCoA, and D) family-level taxonomic summaries of IBS1 culture-independent sample, anaerobic PEA and M9cell cultured communities, sterilized stool and IBS1 DNA plated on PEA or M9cell. Samples were rarified to 18 000 sequences for beta-diversity analyses. E) Taxonomic summaries for IBS1 and the relative abundances of *Dorea* and *Streptococcus* in different cultured communities. PEA – Phenylethyl alcohol agar, cell - cellulose

**Figure S5. Heat map of family-level taxa abundances in each type of media and in culture-independent sequencing of fecal samples.** Each row indicates either a donor fecal sample or a media condition. Each column represents family-level taxa, sorted by phyla. Increasing grades of blue represent greater relative abundance, from 0% to greater than 10%. Refer to Table S1 for media used in this study.

**Figure S6. Unique set of culture conditions required to capture the OTUs from each fecal sample.** Minimum culture conditions required to recover all culturable OTUs (defined as being at least 0.1% relative abundance in plate pools) present at A) greater than 1%, and B) greater than 0.1% relative abundance in culture-independent sequencing of five fecal samples.

**
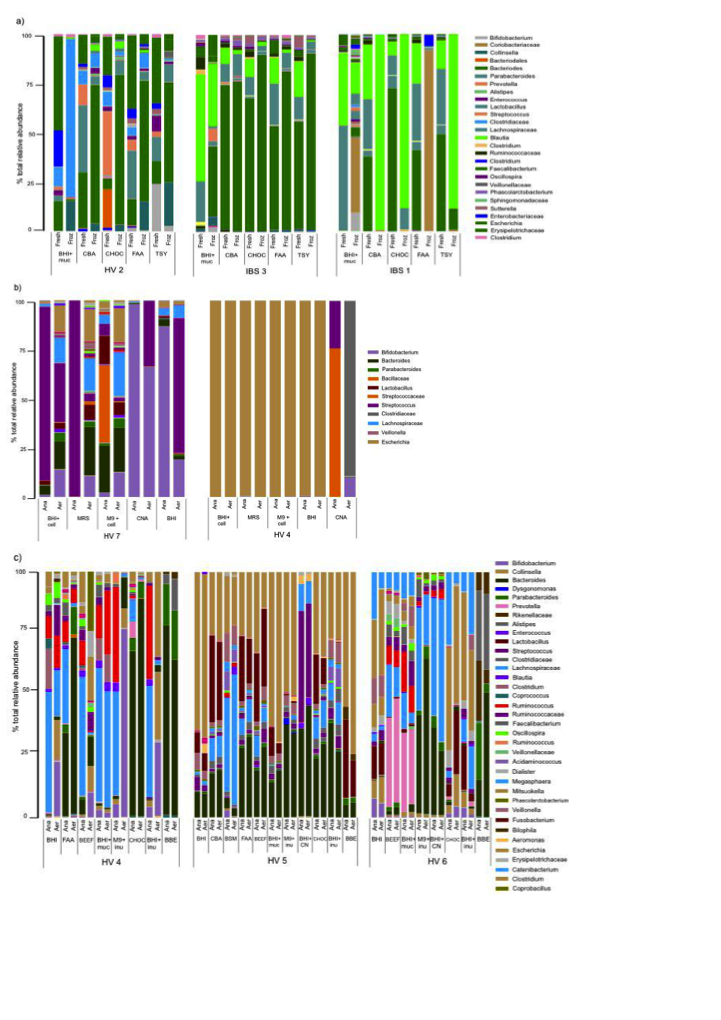
**

**
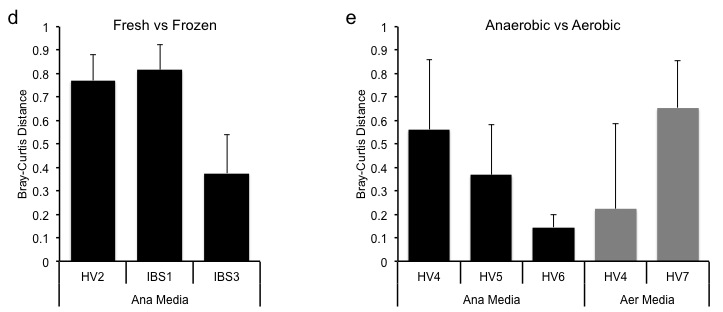
**

**Figure S7. 16S rRNA gene sequencing taxonomic summaries of cultured fresh fecal samples compared to frozen samples, and anaerobic fecal samples compared to aerobic samples.** A) Samples from 3 donors were cultured immediately after collection (Fresh) or after long-term storage at -80^o^C (Frozen). B) Samples from 2 donors were divided into 2 aliquots, one was exposed to oxygen before culturing (Aer) and the other was maintained anaerobic until culturing (Ana) on aerobic media. C) Samples from 3 donors were divided into 2 aliquots, one was exposed to oxygen before culturing (Aer) and the other was maintained anaerobic until culturing (Ana) on anaerobic media. All bacterial groups present at greater than 2% abundance are included in the legend. D) Average Bray-Curtis distances between cultured fecal samples before and after freezing, cultured on 5 types of anaerobic media. 3 samples were cultured immediately after collection or after long-term storage at -80^o^C. All samples rarified to 3932 reads. E) Average Bray-Curtis distances comparing cultured fecal samples, with (Aerobic) or without (Anaerobic) oxygen exposure. Samples were cultured on 5 types of aerobic media and 7 types of anaerobic media. All sample rarified to 8625 reads. Error bars represent S.D. BHI – brain heart infusion, muc – mucin, CBA – Columbia blood agar, CHOC – chocolate agar, FAA – fastidious anaerobe agar, TSY – tryptic soy yeast agar, cell – cellulose, MRS – de Man Rogosa Sharpe agar, CNA – colistin naladixic acid, BEEF – cooked meat agar, inu – inulin, BBE – bacteroides bile esculin agar, CBA – Columbia blood agar, BSM – bifidobacterium selective agar.
